# Integrating microRNA and mRNA expression profiles of neuronal progenitors to identify regulatory networks underlying the onset of cortical neurogenesis

**DOI:** 10.1186/1471-2202-10-98

**Published:** 2009-08-19

**Authors:** Joseph A Nielsen, Pierre Lau, Dragan Maric, Jeffery L Barker, Lynn D Hudson

**Affiliations:** 1 Section of Developmental Genetics, National Institute of Neurological Disorders and Stroke, National Institutes of Health, Bethesda, Maryland, USA; 2 Center for Devices and Radiological Health, Food and Drug Administration, Silver Spring, Maryland, USA; 3 Laboratory of Neurophysiology, National Institute of Neurological Disorders and Stroke, National Institutes of Health, Bethesda, Maryland, USA

## Abstract

**Background:**

Cortical development is a complex process that includes sequential generation of neuronal progenitors, which proliferate and migrate to form the stratified layers of the developing cortex. To identify the individual microRNAs (miRNAs) and mRNAs that may regulate the genetic network guiding the earliest phase of cortical development, the expression profiles of rat neuronal progenitors obtained at embryonic day 11 (E11), E12 and E13 were analyzed.

**Results:**

Neuronal progenitors were purified from telencephalic dissociates by a positive-selection strategy featuring surface labeling with tetanus-toxin and cholera-toxin followed by fluorescence-activated cell sorting. Microarray analyses revealed the fractions of miRNAs and mRNAs that were up-regulated or down-regulated in these neuronal progenitors at the beginning of cortical development. Nearly half of the dynamically expressed miRNAs were negatively correlated with the expression of their predicted target mRNAs.

**Conclusion:**

These data support a regulatory role for miRNAs during the transition from neuronal progenitors into the earliest differentiating cortical neurons. In addition, by supplying a robust data set in which miRNA and mRNA profiles originate from the same purified cell type, this empirical study may facilitate the development of new algorithms to integrate various "-omics" data sets.

## Background

Neurogenesis commences in the developing telencephalon when symmetrically dividing neural stem cells in the neuroepithelium begin to divide asymmetrically [1,2]. Neuronal progenitors proliferate and migrate to form the stratified layers of the cortex, with the earliest neurons forming the preplate or primordial plexiform layer [3,4]. A number of genes that are required for the proper formation of the cortex have already been identified including the transcription factors Pax6 and FoxG1 [5,6]. Larger scale genomic approaches have also been used to identify genes important for cortical development, and these studies have added to the catalog of genes that may be required during cortical neurogenesis [7,8].

Other participants in gene regulatory networks include microRNAs (miRNAs), which are short non-coding RNA molecules that bind to target mRNAs and cause either RNA degradation or translation inhibition (reviewed in [9,10]). miRNAs were originally identified in the regulation of a developmental transition in *C. elegans *[11]. miRNAs are expressed in all tissues, but the brain appears to have the highest diversity of miRNA expression [12]. A number of brain-enriched miRNAs have been identified including miR-9 and miR-124a [12-14]. These two miRNAs have been shown to play a role in promoting the transition from neuronal progenitors into differentiated neurons [15,16]. Several reports have identified inverse expression patterns between a miRNA and predicted targets, including brain-enriched miRNAs [17,18]. Despite this progress, there remains a large gap in our understanding of how the different miRNAs expressed during brain development regulate cortical neurogenesis.

A major limitation in the network analysis of genetic circuits is the availability of mRNA and miRNA profiles from the same cell type. Most of the transcriptome and microRNAome data derive from separate studies, typically drawn from examining whole tissues instead of individual cell types, and are often taken at different developmental time points. The present study overcomes these limitations and provides a robust data set for developing new algorithms to detect modulation of target gene expression by miRNAs. Our objective was to identify miRNA and mRNA expression patterns that may contribute to the earliest stages of neurogenesis. By obtaining expression-profiling data from highly purified neuronal progenitors, we were able to analyze the dynamic global changes in miRNA and mRNA expression that occur at the onset of neurogenesis. The use of fluorescence-activated cell sorting (FACS) and a positive-selection strategy provided the cell purity required for the analysis of neuronal cell populations from a complex tissue, reducing the ambiguity that can be introduced by contaminating cell populations, and eliminated the pitfalls of cell lines and cultured primary cells.

Here we provide evidence that a subset of miRNAs exhibits expression profiles that are negatively correlated with their predicted target mRNAs, and therefore may be part of a gene expression regulatory network that assists in the transition from proliferating neuronal progenitor cells into differentiated neuronal subtypes. In addition, this study identifies a number of candidate transcription factors (TFs) that are expressed in the developing telencephalon and may be important in initiating cortical neurogenesis.

## Results

### FACS isolation of neuronal progenitors from the developing telencephalon

**Figure 1:**
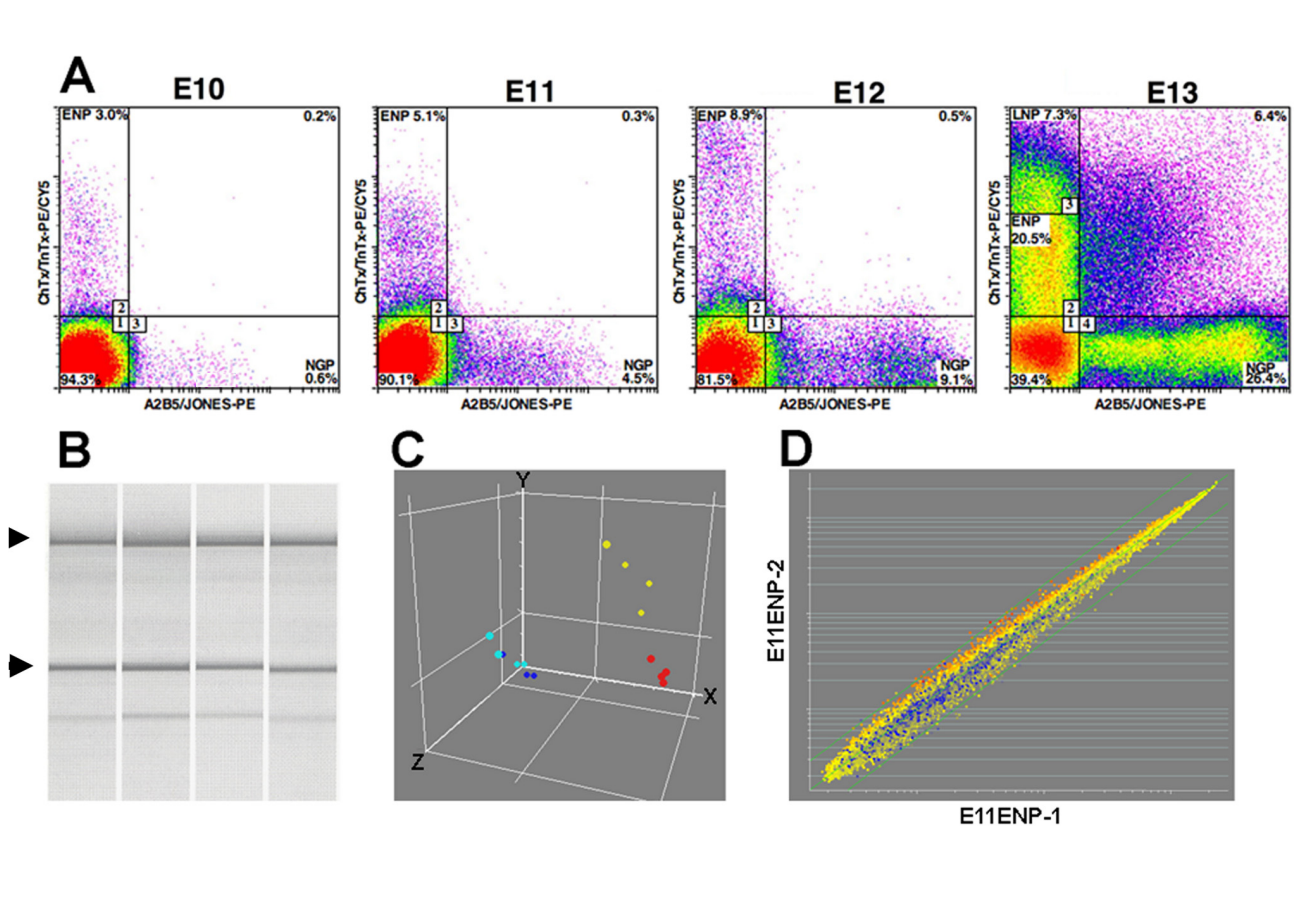
**Neuronal progenitors can be purified directly from the rat telencephalon using flow cytometry**. Dissociates were labeled using the pan-neuronal markers TnTx and ChTx in addition to A2B5 and Jones, which identify a separate lineage that also includes neuronal progenitors and later neuroglial progenitors (NGP). **A**. Bivariate FACS plots reveal a developmental increase in TnTx^+^/ChTx^+^/A2B5^-^/Jones^- ^early neuronal progenitors (ENP) from 3% to 20.5% and the emergence of late neuronal progenitors (LNP) at E13. TnTx^+^/ChTx^+ ^immunofluorescence signals were used to sort-purify populations of viable cells at embryonic days E11, E12 and E13. Sorting gates were set to collect TnTx^+^/ChTx^+^/A2B5^-^/Jones^- ^ENPs and, at E13, LNPs, which were identified on the basis of relative fluorescence intensities of TnTx and ChTx staining reactions (upper left quadrant). **B**. Analysis of total RNA extracted from NPs isolated by FACS shows the presence of intact 18S and 28S rRNA bands (arrow heads). **C**. Principle component analysis demonstrates the reproducibility of the biological replicates. Red circles = E11 ENPs; yellow circles = E12 ENPs; light blue circles = E13 ENPs; dark blue circles = E13 LNPs. **D**. Scatter plot of two E11 ENP biological replicates. Correlation analysis was performed on the biological replicates within each group. The correlation coefficient was greater than 0.94 for each replicate.

We focused on the earliest neuronal progenitors in the rat telencephalon that co-express tetanus toxin and cholera toxin (TnTx^+^/ChTx^+^)-binding gangliosides on their surface membrane, but lack the expression of the neuronal progenitor markers A2B5 and Jones (CDw60), which label a separate pool of progenitors that also generate neurons and later, glial phenotypes. TnTx^+^/ChTx^+^/A2B5^-^/Jones^- ^cells represented 5.1% of the total number of cells in the rat telencephalon at embryonic day 11 (E11), 8.9% at E12, and 27.8% at E13. We have previously defined these TnTx^+^/ChTx^+^/A2B5^-^/Jones^- ^cells as early neuronal progenitors (ENPs) [19]. There were not enough ENPs present at E10, but by E11 these cells could be isolated in sufficient numbers to obtain total RNA for microarray analysis (Figure 1A). At E13, there were enough cells to split the TnTx^+^/ChTx^+^/A2B5^-^/Jones^- ^cells into approximately equal low and high expressing populations, with the latter being defined as late neuronal progenitors (LNPs), based on their later ontogenetic appearance at E13 and progressive accumulation of these cells during late stages of cortical neurogenesis [20]. The positively-selected ENPs and LNPs cells were sorted directly into RNAlater to preserve RNA integrity. The quality of the total RNA isolated with this method was confirmed by the presence of intact ribosomal RNA bands (Figure 1B). After hybridization, the microarray dataset was assessed using principle component analysis. The independent biological replicates clustered together by embryonic days of development (Figure 1C). There was little difference in the expression profiles of ENPs and LNPs at E13, as these two populations did not resolve into separate groups. Correlation analysis was performed on each group of biological replicates (Figure 1D). The correlation coefficient was greater than 0.94 for the biological replicates at each of the developmental time points, demonstrating a high level of reproducibility among independently obtained replicates.

**Figure 2:**
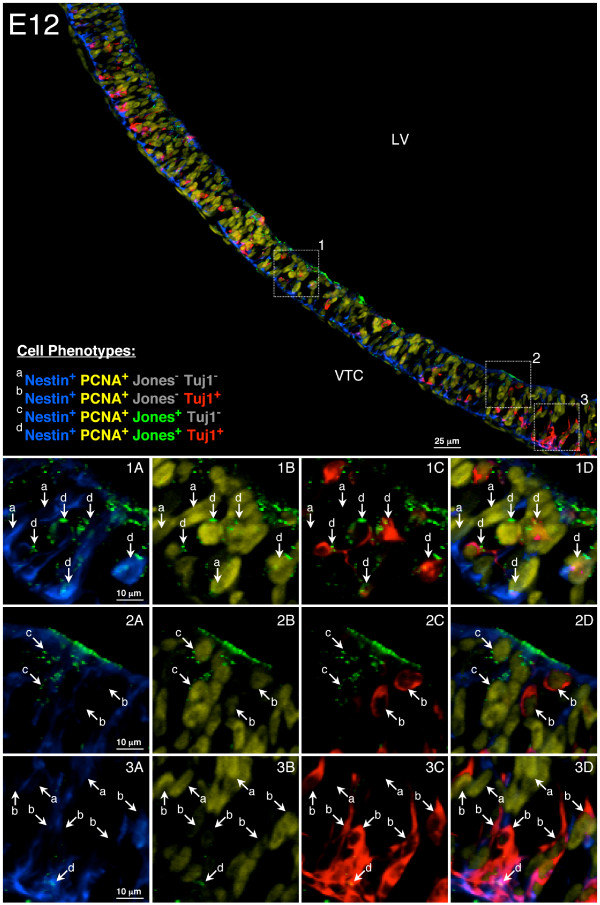
**Neuronal progenitors are located in the ventral telencephalon near the pial surface**. Sagittal sections of E12 rat ventral telencephalon were immunostained with anti-nestin (blue), anti-PCNA (yellow), anti-CDw60/Jones (green) and anti-beta-tubulin III/Tuj1 (red). Tuj1 antibody was used in place of TnTx to provide a better visualization of neuronal cell bodies. Insets 1–3 in the top panel are enlarged in the bottom panels (1A-D, 2A-D, 3A-D) to show the anatomical distribution of the 4 major neural phenotypes, which were classified as follows: (a) Nestin^+^PCNA^+^Jones^-^Tuj1^- ^neural stem/precursor cells, (b) Nestin^+^PCNA^+^Jones^-^Tuj1^+ ^neuronal progenitors and post-mitotic neurons, which are predominantly Nestin^-^PCNA^-^, (c) Nestin^+^PCNA^+^Jones^+^Tuj1^- ^neuroglial progenitors and (d) Nestin^+^PCNA^+^Jones^+^Tuj1^+ ^neuronal progenitors, The majority of Jones^-^TnTx^+ ^neuronal progenitors isolated by FACS for microarray analysis in this study are represented by Jones^-^Tuj1^+ ^cells (population b) located near the pial surface as shown in panels 3C and 3D.

To provide spatial information on the neuronal progenitor cell population in the developing telencephalon, sagittal sections of E12 rat ventral telencephalon were immunostained with anti-nestin, anti-PCNA, anti-CDw60/Jones and anti-beta-tubulin III/Tuj1. Tuj1 (a pan-neuronal cytoskeletal marker) was used in place of TnTx (a pan-neuronal surface marker) to provide better visualization of neuronal cell bodies in the section. Four different cell phenotypes were identified using this method (Figure 2). The Jones^-^/TnTx^+ ^neuronal progenitors isolated by FACS for microarray analysis at E12 in this study are represented by Jones^-^/Tuj1^+ ^cells (population b in Figure 2, panels 3C and 3D), which are predominately located near the pial surface of the ventral telencephalon.

### mRNAs and miRNAs are dynamically regulated during neurogenesis

**Figure 3:**
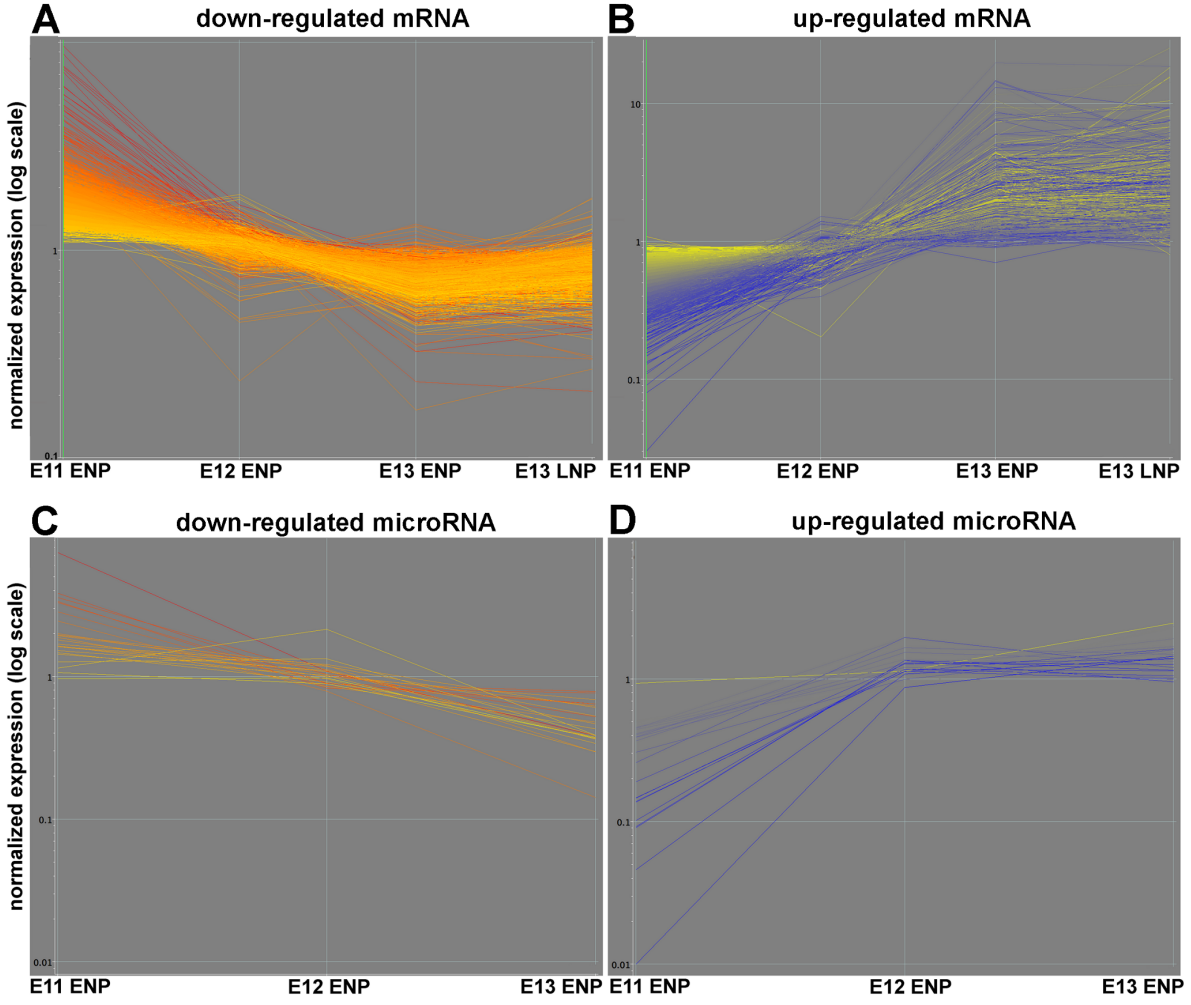
**mRNAs and miRNAs are dynamically regulated during the onset of cortical neurogenesis. K-means clustering analysis was applied to the normalized expression data (see Materials and Methods)**. Two major classes of differentially regulated mRNAs (**A, B**) and miRNAs (**C, D**) were identified. The mRNA and miRNA expression levels were colored by the relative expression levels in E11 ENPs with red being the highest and blue being the lowest globally normalized expression level.

Clustering analysis was used to identify the prevalent gene expression patterns in the data sets. In the mRNA microarray experiment, there was little change in the expression level of the vast majority of mRNAs represented by the 31,099 probe sets on the Affymetrix microarrays during the developmental period surveyed. Only 7.2% of the probe sets underwent significant changes between E11 to E13. The gene lists were obtained using a combination of a statistical filter and a 2-fold change in normalized expression value (see Methods section). There were two dominant gene expression patterns for these mRNAs. One class of mRNAs was down-regulated as development progressed from E11 to E13 (Figure 3A), while another class of mRNAs was up-regulated during this time frame (Figure 3B). A parallel result came from the clustering analysis of differentially-regulated miRNAs. One class of miRNAs was down-regulated and another class up-regulated between E11 and E13 (Figure 3C, D). There were 21 miRNAs down-regulated and 11 miRNAs up-regulated between E11 and E13 (Table 1). Among the miRNAs identified as up-regulated, there were several that have previously been found to be expressed in neuronal progenitors, including miR-9 and miR-124a. As was the case for mRNAs, the majority of miRNAs did not change expression patterns from E11 to E13.

**Table 1:** Differentially regulated miRNAs identified in neuronal progenitors between E11 and E13 using microarray analysis.

**Down-regulated**	**FoldΔ**	**Up-regulated**	**FoldΔ**
miR-292-3p	19.2	miR-9	144.3
miR-126	17.1	miR-125b	30.3
miR-200c	6.8	miR-125a	12.7
miR-20b*	6.6	miR-181b	12.4
miR-291-3p	6.0	miR-99a	10.3
miR-20b	5.5	miR-100	7.2
miR-363-3p	5.2	miR-99b	7.2
miR-199a	4.4	miR-181c	7.2
miR-145	4.4	miR-218	4.6
miR-183	4.2	miR-7	4.1
miR-143	4.2	miR-124a	2.6
miR-92	4.2		
miR-200b	3.9		
miR-19a	3.5		
miR-222	3.3		
miR-205	3.2		
miR-18	3.1		
miR-219	2.9		
miR-210	2.8		
miR-214	2.6		
miR-290	2.6		

**Figure 4:**
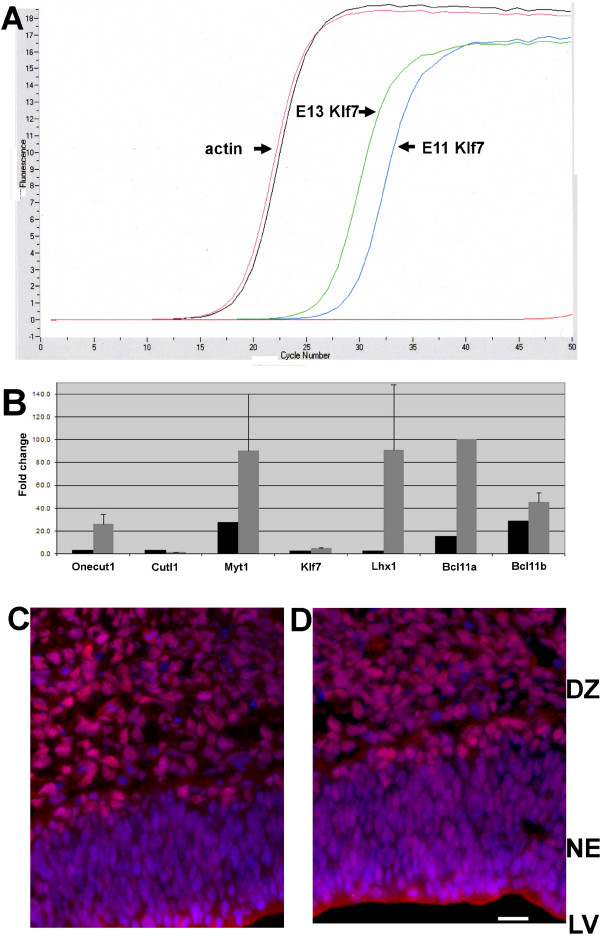
**Differentially expressed mRNAs detected by microarrays were validated with qRT-PCR and immunohistochemistry**. **A**. The cycle threshold of Klf7 is shifted to the left in E13 ENPs confirming an increase in Klf7 mRNA expression level, while the beta-actin mRNA expression level remains nearly constant. **B**. The fold changes from E11 to E13 for seven transcription factors identified by microarrays (black bars) and qRT-PCR (grey bars) are plotted. **C, D**. Cutl1 protein (red fluorescence) is expressed at E13 in the differentiating zone (DZ) of the ventral (**C**) and dorsal (**D**) telencephalon. The blue fluorescence depicts nuclear counterstain with DAPI. The lateral ventricle (LV) is at bottom of the figure. NE = neuroepithelium. Scale bar = 20 microns.

Before proceeding with a bioinformatic analysis of the datasets, microarray validation was performed using quantitative reverse transcription-PCR (qRT-PCR) and immunohistochemistry. qRT-PCR was first used to validate miRNA expression, and the data confirmed the expression of each of the 11 miRNAs identified in E13 ENPs (Table 1, see additional file 1). To validate the mRNA microarray data, qRT-PCR was used to detect 7 TFs that were up-regulated in ENPs between E11 and E13, including *onecut, Cutl1, Myt1, Klf7, Lhx1, Bcl11a *and *Bcl11b *(Figure 4A, B). The qRT-PCR results agreed with the microarray data for each of the 7 TFs tested. The magnitude of the fold-changes differed, but this likely reflects the difficulty in reproducibly detecting low expression levels of transcripts at E11. An antibody directed against the Cutl1 protein was used to confirm the protein expression of one of the TFs identified by microarray analysis. Cutl1 protein expression was detected in the ventral and dorsal telencephalon consistent with the 3.0-fold increase in Cutl1 mRNA expression in E13 ENPs (Figure 4B, C, D). Additional validation was obtained using the Mahoney transcription factor *in situ *hybridization database [21]. In total, sixty TFs were identified as up-regulated in E13 ENPs (Table 2). Of those 60 TFs, 30 were found in the Mahoney database at mouse E13, which is approximately 1.5 days earlier than the rat E13 time-point used in this study. Notably, 16 of 30 (53%) displayed a positive *in situ *hybridization signal in the mouse telencephalon.

**Table 2:** 60 mRNAs encoding transcription factors are up-regulated in neuronal progenitors between E11 and E13.

**Affymetrix ID**	**FoldΔ**	**GenBank**	**Gene**	**Gene Description**
1379530_at	48.3	AA964658		Eomesodermin
1392064_at	30.2	BF400590	Dlx1	distal-less homeobox 1 (predicted)
1383010_at	19.1	AW531880		B-cell CLL/lymphoma 11A (zinc finger protein) (predicted)
1377171_at	10.9	AA875041	Lzts1	leucine zipper, putative tumor suppressor 1
1391948_at	9.8	BM390227	Bcl11b	B-cell leukemia/lymphoma 11B (predicted)
1381350_at	9.8	AI317824		Transcribed locus
1387288_at	9.5	NM_019218	Neurod1	neurogenic differentiation 1
1369679_a_at	9.2	AB060652	Nfia	nuclear factor I/A
1378776_at	6.8	BE097103	Pou6f1	POU domain, class 6, transcription factor 1
1374625_at	6.6	AI176616	Hes6	hairy and enhancer of split 6 (Drosophila) (predicted)
1394022_at	6.2	BE116009	Idb4	inhibitor of DNA binding 4
1371570_at	6.0	AI406266	Scrt1	scratch homolog 1, zinc finger protein (predicted)
1380552_at	6.0	AI555855		Transcribed locus
1374187_at	5.9	AI233361		Transcribed locus
1371202_a_at	5.9	AB012232	Nfib	nuclear factor I/B
1383937_at	5.6	BI284495		transcription factor AP-2, gamma
1367946_at	4.7	NM_017365	Pdlim1	PDZ and LIM domain 1 (elfin)
1379674_at	4.5	BE115481	Cbfa2t3	core-binding factor, alpha subunit 2;3 (predicted)
1371043_a_at	3.9	BE107327	Pou3f3	POU domain, class 3, transcription factor 3
1383573_at	3.9	BE117444		Serologically defined colon cancer antigen 33 (predicted)
1377206_at	3.6	BI282093	Lhx1	LIM homeobox protein 1
1387349_at	3.4	NM_013028	Shox2	short stature homeobox 2
1369765_at	3.3	NM_022384	Ascl1	achaete-scute complex homolog-like 1 (Drosophila)
1388185_at	3.2	AI178012	Rb1	retinoblastoma 1
1370946_at	3.2	BF420722	Nfix	nuclear factor I/X
1370963_at	3.2	AJ131902	Gas7	growth arrest specific 7
1385173_at	3.1	BE116944	Ebf3	early B-cell factor 3 (predicted)
1370694_at	3.1	AB020967	Trib3	tribbles homolog 3 (Drosophila)
1378859_at	3.0	AI058446		aristaless 3
1372299_at	3.0	AI013919	Cdkn1c	cyclin-dependent kinase inhibitor 1C (P57)
1371024_at	3.0	AW527515	Cutl1	cut-like 1 (Drosophila)
1398548_at	2.9	BF389746	Nr6a1	Nuclear receptor subfamily 6, group A, member 1
1371034_at	2.9	BF396189	Onecut1	one cut domain, family member 1
1387122_at	2.9	NM_012760	Plagl1	pleiomorphic adenoma gene-like 1
1378487_at	2.7	BF403191	Ep300	E1A binding protein p300
1384840_at	2.7	AA924754	Prrx1	Paired related homeobox 1
1395161_at	2.7	AW531604	Myt1	myelin transcription factor 1 (predicted)
1387200_at	2.6	NM_021770	Olig1	oligodendrocyte transcription factor 1
1392089_at	2.6	AW435314	Hcfc2	host cell factor C2 (predicted)
1392477_at	2.6	AI059914	Etv1	ets variant gene 1 (predicted)
1388701_at	2.5	AI555336		mixed lineage-leukemia translocation to 6 homolog
1372274_at	2.5	AI009727		mixed-lineage leukemia 5 (trithorax homolog)
1383247_a_at	2.5	BI291029	Mybbp1a	MYB binding protein (P160) 1a
1387274_at	2.5	NM_012943	Dlx5	distal-less homeobox 5
1372093_at	2.5	AI409308	Mxi1	Max interacting protein 1
1383736_at	2.4	AI145457	Elavl2	ELAV (embryonic lethal, abnormal vision)
1397779_at	2.4	BF562254		Chromodomain helicase DNA binding protein 2 (predicted)
1383166_at	2.4	BE100595		Transcribed locus
1380363_at	2.4	BF420490	Klf7	Kruppel-like factor 7 (ubiquitous) (predicted)
1386080_at	2.4	BE107815	Hey1	Hairy/enhancer-of-split related with YRPW motif 1
1385227_at	2.4	BF398245	Trps1	trichorhinophalangeal syndrome I (predicted)
1385215_at	2.3	BF388857	Cbfa2t1	core-binding factor, runt domain, alpha subunit 2
1372319_at	2.3	BF282811		Transcribed locus, strongly similar to XP_230811.3
1392676_at	2.2	AI548984		myocardial ischemic preconditioning upregulated 1
1370535_at	2.2	U48809	Myt1l	myelin transcription factor 1-like
1397734_at	2.2	AW524780		Sp3 transcription factor
1368841_at	2.1	NM_053369	Tcf4	transcription factor 4
1373256_at	2.1	BM391119	Chd3	Chromodomain helicase DNA binding protein 3 (predicted)
1389994_at	2.1	BE104268	Sox11	SRY-box containing gene 11
1381489_at	2.1	BE114007	Sox6	SRY-box containing gene 6 (predicted)

**Figure 5:**
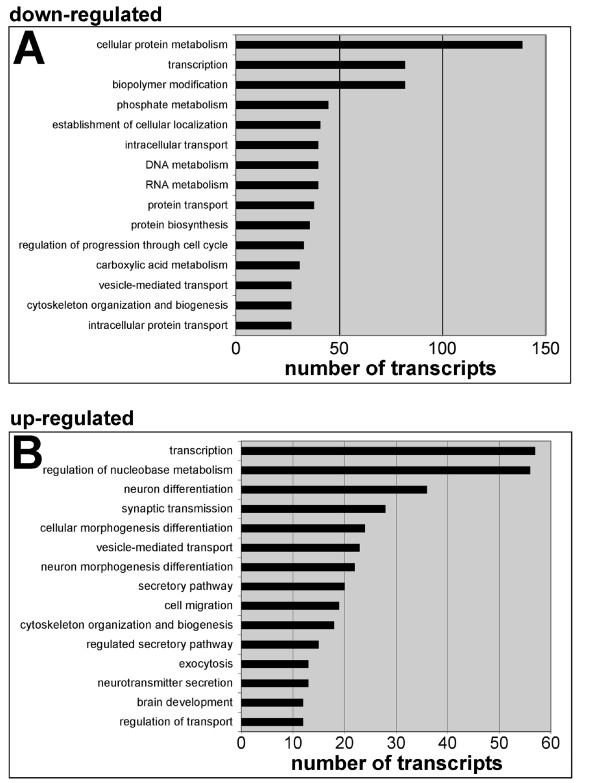
**Gene ontology (GO) analysis of differentially regulated transcripts**. The mRNAs encoding transcription factors make up the second largest class of mRNAs being down-regulated (**A**) and the largest class of mRNAs being up-regulated (**B**). Differentially regulated gene lists (see methods) were imported into DAVID for GO analysis. The analysis was confined to the biological process category (GO level 5). The 15 GO categories with the highest number of mRNAs annotated were included with the number of mRNAs in each category graphed on the x-axis.

After validation of the microarray dataset, gene ontology (GO) analysis was performed to identify the classes of genes that were being differentially regulated in ENPs between E11 and E13. The 15 biological process GO categories with the most mRNAs in both the down-regulated class (Figure 5A), and the up-regulated class (Figure 5B) are detailed in Figure 5. The most striking finding was the number of mRNAs encoding TFs that were identified in both categories of differentially regulated genes (Table 2 and see additional file 2). Also of note was the number of cell cycle genes that were being down-regulated (see additional file 3), and the large number of up-regulated transcripts encoding proteins important for neuronal differentiation (Table 3).

**Table 3:** Gene ontology analysis identifies 35 transcripts up-regulated in E13 neuronal progenitors annotated with the GO term: neuron differentiation.

**Affymetrix ID**	**FoldΔ**	**GenBank**	**Gene**	**Gene Description**
1384944_at	43.3	BE116855	Bcl11b	B-cell leukemia/lymphoma 11B (predicted)
1370041_at	11.7	NM_053440	Stmn2	stathmin-like 2
1378776_at	6.8	BE097103	Pou6f1	POU domain, class 6, transcription factor 1
1368411_a_at	4.6	X74211	Mtap2	microtubule-associated protein 2
1391019_at	3.4	BF285698	Slitrk1	SLIT and NTRK-like family, member 1 (predicted)
1377308_a_at	3.4	BF398408		Neurotrophic tyrosine kinase, receptor, type 3
1368097_a_at	3.4	NM_053865	Rtn1	reticulon 1
1369765_at	3.3	NM_022384	Ascl1	achaete-scute complex homolog-like 1 (Drosophila)
1371618_s_at	3.3	AI229029	Tubb3	tubulin, beta 3
1370043_at	3.3	NM_031753	Alcam	activated leukocyte cell adhesion molecule
1372299_at	3.0	AI013919	Cdkn1c	cyclin-dependent kinase inhibitor 1C (P57)
1368261_at	2.7	NM_053817	Nrxn3	neurexin 3; hypothetical gene supported by NM_053817
1395357_at	2.7	BG672052	Map1b	microtubule-associated protein 1b
1390671_at	2.7	AI044666	Igf1r	Insulin-like growth factor 1 receptor
1373683_at	2.7	AI230396	Fyn	fyn proto-oncogene
1387200_at	2.6	NM_021770	Olig1	oligodendrocyte transcription factor 1
1392477_at	2.6	AI059914	Etv1	ets variant gene 1 (predicted)
1367918_at	2.5	NM_031066	Fez1	fasciculation and elongation protein zeta 1 (zygin I)
1387274_at	2.5	NM_012943	Dlx5	distal-less homeobox 5
1388015_at	2.4	U04998	Ptprz1	protein tyrosine phosphatase, receptor-type, Z polypept. 1
1379693_at	2.4	AI409154	Robo2	Roundabout 2 (Drosophila)
1395986_at	2.3	BF391439	Slit2	slit homolog 2 (Drosophila)
1388419_at	2.3	AW915005		CDK5 regulatory subunit associated protein 2
1369213_at	2.3	NM_017345		neural cell adhesion molecule L1
1379526_at	2.3	BG374506	Mbp	Myelin basic protein
1387424_at	2.2	NM_012884	Cntn2	contactin 2
1370124_at	2.2	NM_053968	Mt3	metallothionein 3
1368148_at	2.2	NM_012610	Ngfr	nerve growth factor receptor (TNFR superfamily, mem 16)
1381215_at	2.2	AA900967		PREDICTED: similar to Numbl protein [Rattus norvegicus]
1368472_at	2.1	NM_031320	Celsr3	cadherin EGF LAG seven-pass G-type receptor 3
1396150_at	2.1	AW917275	Cldn1	claudin 1
1374403_at	2.1	BF412072	Efnb1	ephrin B1
1382205_at	2.1	AW527509		Transcribed locus
1386948_at	2.0	NM_012987	Nes	Nestin
1368879_a_at	2.0	AF413212	Gnao	guanine nucleotide binding protein, alpha o

### miRNA expression is negatively correlated with predicted target mRNA expression

**Figure 6:**
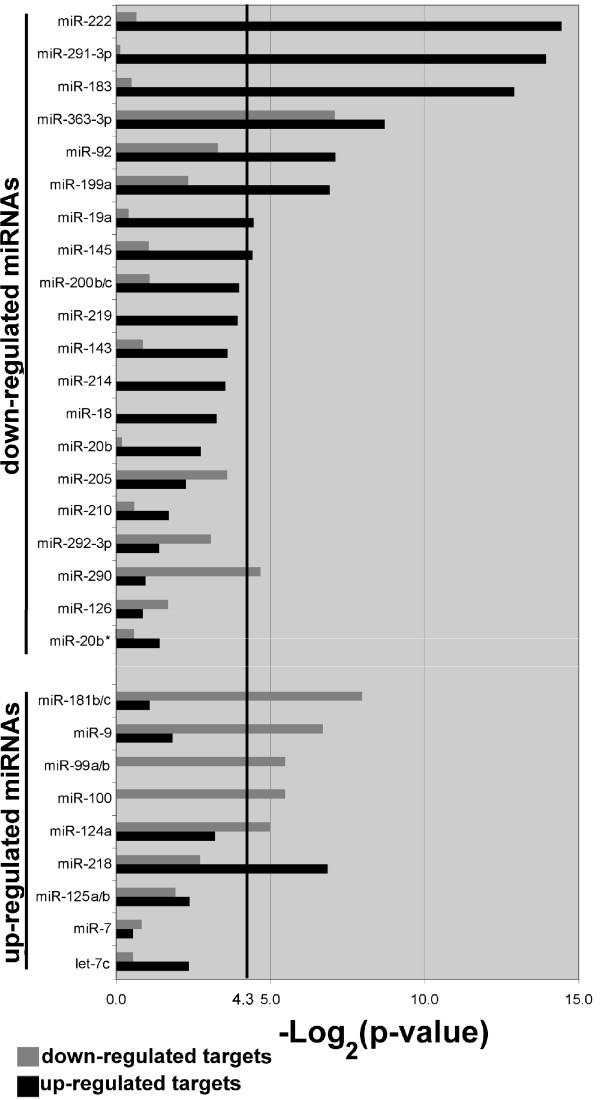
**miRNA expression is negatively correlated with target mRNA expression**. Predicted target mRNAs for each miRNA were identified using TargetScan 4.0 and compared with the lists of experimentally-determined, differentially-regulated mRNAs. A two-tailed Fisher's Exact Test was used to determine whether there were more predicted target mRNAs with differential expression than would be expected by chance (p < 0.05, above heavy black line at 4.3). The negative log of the p-value is plotted on the x-axis for both down-regulated mRNAs (grey) and up-regulated mRNAs (black).

In addition to the identification of genes that may be important during neurogenesis, the mRNA and miRNA datasets were compared to determine whether there was evidence of differential expression of miRNAs influencing mRNA expression during the onset of cortical neurogenesis. The predicted targets for each of the miRNAs that were differentially regulated were compiled using TargetScan 4.0. The predicted mRNA targets were compared to the experimentally-determined, differentially-regulated mRNAs to identify the number of predicted mRNA targets that actually changed expression levels between E11 and E13. A two-tailed Fisher's Exact Test was used to determine whether the number of target mRNAs that changed expression was greater than would be expected by chance. The results of this analysis are presented in Figure 6. When testing miRNAs that were down-regulated, 7 of 21 miRNAs (33%) had a significant number of target mRNAs that were up-regulated. miR-199a had significantly less target mRNAs up-regulated than would be expected by chance, and was not counted as having an inverse expression pattern. When testing miRNAs that were up-regulated, 7 of 11 miRNAs (64%) had a significant number of mRNA targets that were down-regulated.

**Figure 7:**
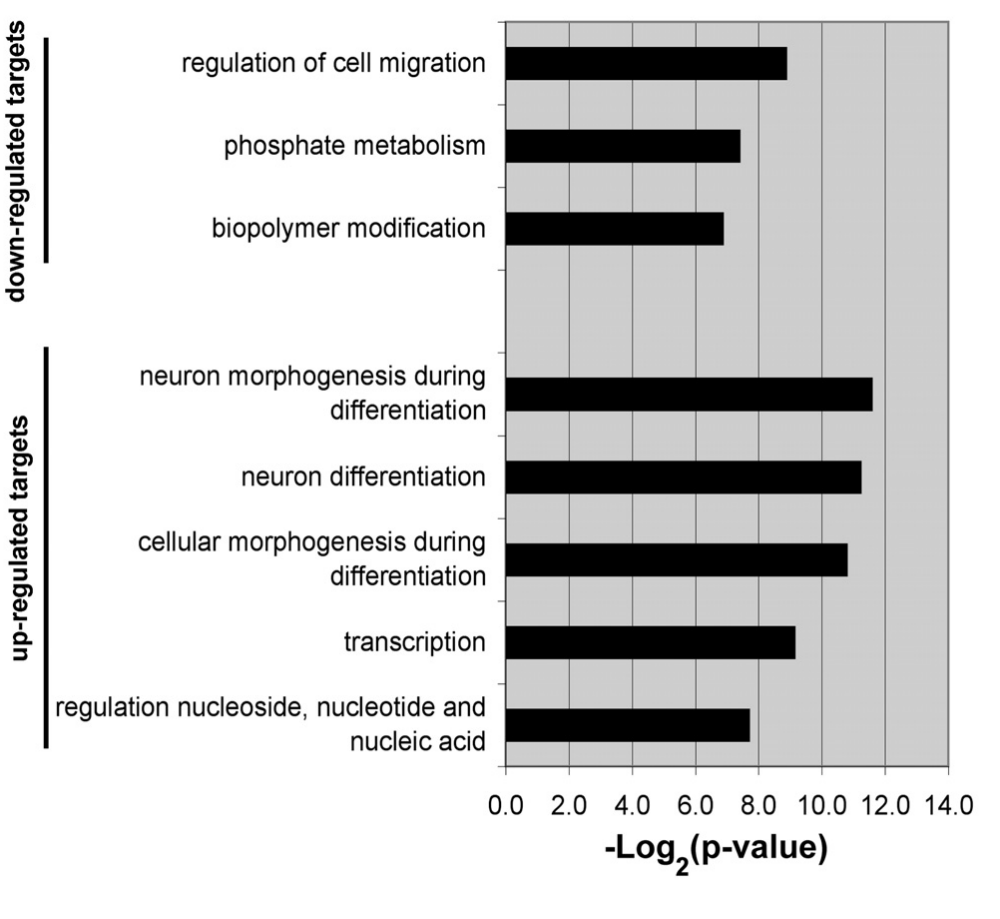
**GO analysis identifies potential mRNA functional categories regulated by miRNAs during neuron differentiation**. GO analysis was performed on mRNAs predicted to be targets of miRNAs, which were negatively correlated with target mRNA expression. The GO analysis was confined to the biological process category (GO level 5). Only categories with a p-value greater that 0.05 are included. The negative log of the p-value is plotted on the x-axis.

GO analysis was performed on the list of predicted target mRNAs that were in common with mRNAs that were differentially regulated between E11 and E13. This analysis compared the mRNAs that were predicted to be targeted by miRNAs with those mRNAs that were experimentally determined to change expression levels. Cell migration was the top GO category of down-regulated mRNAs predicted to be targeted by up-regulated miRNAs. Moreover, genes regulating neuron differentiation was the top GO category of up-regulated mRNAs predicted to be targeted by down-regulated miRNAs (Figure 7).

**Figure 8:**
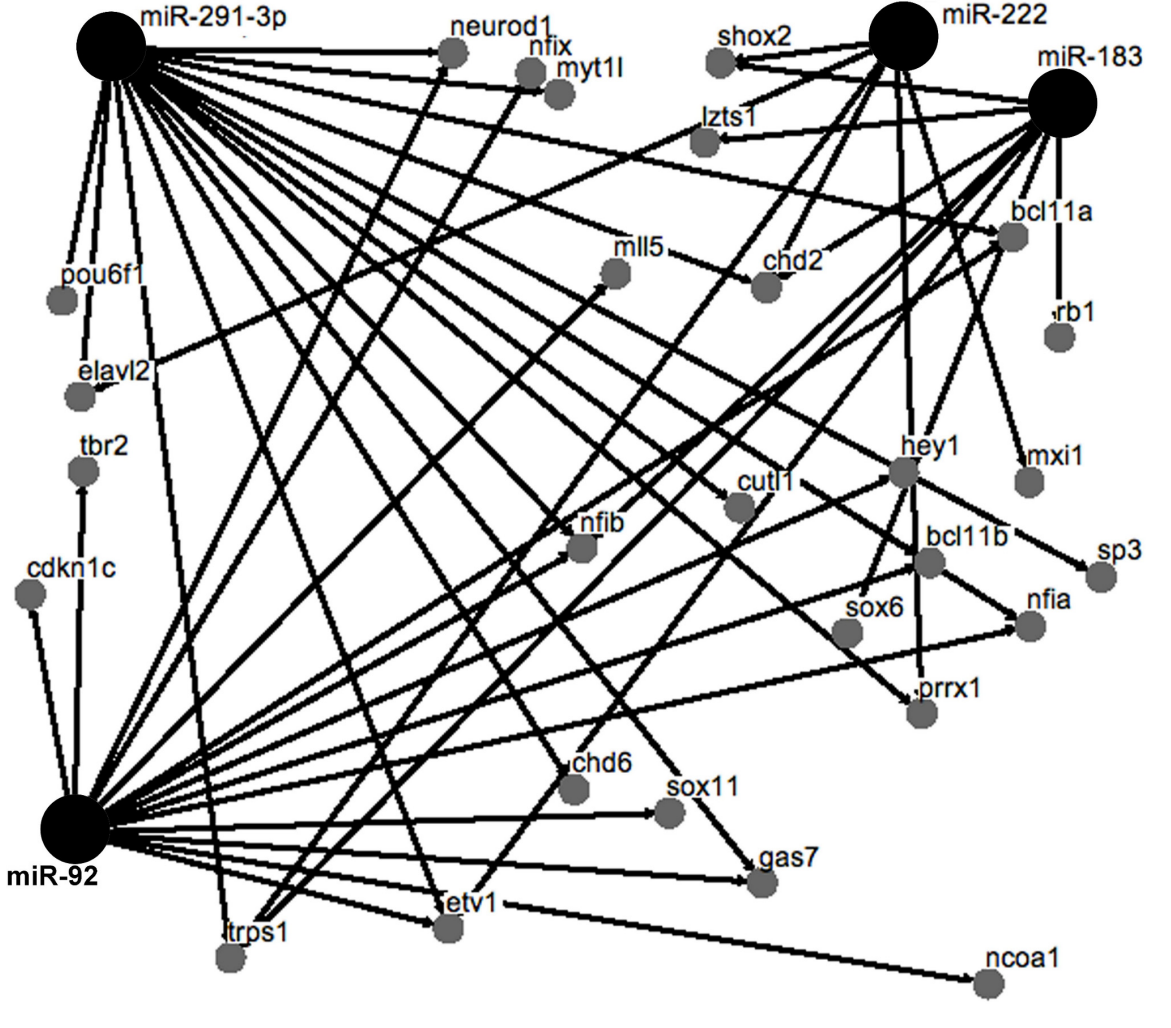
**Network analysis identifies miRNAs that are predicted to target multiple transcription factors**. The small grey circles identify TFs that were up-regulated in neuronal progenitors between E11 and E13. Larger black circles identify miRNAs that were down-regulated in neuronal progenitors between E11 and E13. The connection lines indicate that a miRNA binding site was identified in the 3' UTR of the transcription factor using TargetScan for miRNA target prediction.

Network analysis was performed to determine whether different miRNAs were predicted to interact with common target mRNAs encoding TFs. We used TargetScan 4.0 to predict miRNA binding sites in the 3' untranslated region (3'UTR) of the 60 up-regulated TFs in E13 ENPs. There were 74 predicted miRNA binding sites for miRNAs that were down-regulated. There were 55 miRNA predicted binding sites for miRNAs that were up-regulated. Focusing on the down-regulated miRNAs that had a significant negative correlation in Figure 6, we performed network analysis and identified miR-92, miR-183, miR-222 and miR-291-3p as predicted to target multiple TFs that were up-regulated in E13 ENPs (Figure 8).

## Discussion

Neurogenesis is an extraordinarily complex process that is just beginning to be understood at the molecular level. In this study, we sought to identify genes that are important in regulating the onset of cortical neurogenesis, and to determine whether specific miRNAs may be contributing to gene regulation during this developmental period. Key to our approach was purifying neuronal progenitor cell populations at the earliest stages of cortical neurogenesis using FACS and obtaining the transcriptome and miRNAome profiles of these cells. This study extends our previous work on lineage-negative NSCs [19], to now include the positively selected neuronal progenitor progeny of NSCs. In the current study, we included two additional lineage-restricted markers (TnTx and A2B5) to ChTx and Jones used in our previous neural cell identification strategy. TnTx is a pan-neuronal surface marker that labels early neuronal progenitors and post-mitotic neurons. A2B5 is a neuroglial progenitor marker that is retained by early neuronal populations specifically derived from this progenitor (see reference [20]). TnTx and A2B5 have largely overlapping expressions with ChTx and Jones, respectively. All four markers were used in this study to more stringently discriminate and isolate by FACS the TnTx+/ChTx+ NPs derived from A2B5-Jones-precursors, which predominate at the onset of cortical neurogenesis, and to minimize contamination from TnTx+/ChTx+ NPs derived from A2B5+Jones+ progenitors that increasingly emerge during later cortical development. Four independent biological replicates were collected for each time-point with very good reproducibility. Using this "-omics" strategy, we identified two major classes of dynamically regulated mRNAs and miRNAs between embryonic days E11 and E13, a developmental period that spans the onset of rat cortical neurogenesis.

The GO analysis focused our attention on TFs since mRNAs encoding TFs make up the largest class of genes being up-regulated and the second largest class of genes being down-regulated in the dataset. The high number of TFs up-regulated (60 TFs listed in Table 2) was not surprising considering the likely heterogeneity of neuronal subpopulations to emerge by E13 in the telencephalon. At this stage of development, the telencephalon includes: pioneer neurons, Cajal-Retzius neurons, tangentially migrating GABAergic neurons, and some of the first pyramidal neurons [2,3]. Likewise, a large number of TFs (83 listed in additional file 2) were down-regulated between E11 and E13. We speculate that the diversity of TFs in the proliferating E11 neuronal progenitor population may arise from multiple TF expression domains along the rostral-caudal axis of the telencephalon. Summary of all the TFs identified here is beyond the scope of this paper, but up-regulated TFs include proteins with previously defined roles in cortical development. For example, the transcription factor Bcl11b is up-regulated 28.6-fold in E13 ENPs and is required for proper axonal projection of corticospinal motor neurons [22]. Dlx1 is up-regulated 30.2-fold in E13 ENPs, and in transgenic mice lacking both Dlx1 and Dlx2, GABAergic interneurons in the forebrain fail to migrate [23]. Klf7 is up-regulated 2.4-fold in E13 ENPs, and Klf7 null mice show a reduction in cortical thickness [24]. Overall, these results suggest that the novel TFs identified in this study may also be important in regulating cortical development.

miRNAs have proven to be important in a number of different developmental processes including the regulation of neuronal progenitors progressing into differentiated neurons [15,16]. In this study, we contribute to the catalog of miRNAs that are expressed during mammalian brain development. The developmental time course of this experiment allowed the identification of differentially-expressed miRNAs during the earliest stages of cortical neurogenesis between E11 and E13.

The expression profiles of both mRNAs and miRNAs allowed us to ask whether there was a correlation between the expression levels of miRNAs and the predicted mRNA targets of each miRNA. We found that 33% of the miRNAs that were down-regulated and 64% of the miRNAs that were up-regulated were indeed negatively correlated with the expression pattern of their predicted target mRNAs. Notably, we found miR-222, miR-291-3p, miR-183, miR-363-3p, miR-92, miR-19a and miR-145 as down-regulated miRNAs between E11 and E13 and whose expression was negatively correlated with the expression of their predicted targets. No clear roles for these miRNAs have been established yet. Of interest on the list of miRNAs that were down-regulated between E11 and E13 was the miR-290 family, which is expressed by embryonic stem (ES) cells [25,26]. The number of mRNAs down-regulated between E11 and E13 encoding proteins involved in cell cycle regulation (see additional file 3), and the expression of miR-290, miR-291-3p, and miR-292-3p, are interpreted as an indication of the proliferative phenotype of the E11 ENPs, which likely contain cells that are only a cell division away from neural stem cells. We also found up-regulated miRNAs between E11 and E13 belonging to a well-characterized category of brain-enriched miRNAs. These include miR-7, miR-9, miR-124a, miR-125a/b, miR-181b/c and miR-99a/b. Interestingly, miR-9, miR-124a, miR-99a/b and miR-181b/c up-regulation during early cortical neurogenesis was negatively correlated with the expression level of their predicted targets, consistent with reports that have found a negative expression correlation between tissue-enriched miRNAs and their putative targets [17,18]. Some brain-enriched miRNAs have also been reported to be co-expressed with their target genes [27]. The complexity of the mammalian nervous system may give rise to these disparate findings, which could be caused by differences in developmental time points examined or differences in cell types used in the analysis.

Currently, mammalian miRNAs are not thought to be a primary gene regulatory mechanism. Rather, mammalian miRNAs are thought to act more by fine-tuning the changes of gene expression that are largely under transcriptional control [28]. We speculate that the negative correlation between miRNAs expression and the predicted target mRNA expression identified in this study may be an indication that these tissue enriched miRNAs help to fine-tune transcriptional control by post-transcriptionally modulating the levels of their target mRNAs. Experimental validation of miRNA-target mRNA interaction is required to confirm this hypothesis.

The mechanisms by which miRNAs degrade their targets are not well characterized. There may be cooperation between translation inhibition and mRNA degradation involving deadenylase and decapping complexes [29]. In a rare example in which miRNA directs slicer-mediated cleavage of the target, a high degree of complementarity exists between the miRNA and its binding site in the 3'UTR [30]. Since mammalian miRNAs interact with their targets using less extensive base pairing in the binding site, our data support the fine-tuning model for tissue-enriched miRNAs and leave open the possibility that miRNAs employ mRNA degradation more often than currently recognized, through yet uncharacterized mechanisms.

The negative correlation between miRNAs and predicted target mRNAs expression identified in this study supports the hypothesis that miRNAs may be modulating the expression of genes required during cortical neurogenesis. Further support for this hypothesis was provided by the GO analysis of the differentially regulated mRNAs that were classified as predicted targets of miRNAs. Notably, mRNAs that were down-regulated and predicted to be targeted by up-regulated miRNAs were enriched in mRNAs encoding proteins involved with cell migration (Figure 7). One of these mRNAs was *Cxcr4*, which is down-regulated 2.4-fold and is predicted to be targeted by miR-9, which was up-regulated over a hundred fold. Since CXCR4 is required for radial and tangential migration in the developing cortex [31], we speculate that the up-regulated miRNAs identified in this study may help dampen the expression of genes no longer required as neuronal differentiation progresses, as has been previously suggested [10,32].

The mRNA encoding the cyclin dependent kinase inhibitor 1c (*Cdkn1c*) was up-regulated 3-fold in E13 ENPs and is predicted to be targeted by three miRNAs that were down-regulated: miR-92, miR-222, and miR-363-3p. CDKN1C has been reported to negatively regulate proliferation of cortical progenitors by inhibiting the cell cycle progression machinery [33]. It is thus tempting to hypothesize that the down-regulated miRNAs identified in this study may act as progenitor maintenance factors helping to prevent aberrant or leaky expression of genes that would interfere with the proper timing of cortical neurogenesis. Additional support for this hypothesis was provided by the miRNA-TF mRNA network analysis in Figure 8. We found four down-regulated miRNAs (miR-92, miR-183, miR-222 and miR-291-3p) that were predicted to target multiple TFs, which were up-regulated in E13 ENPs. The importance of keeping pro-neural TFs silenced in neuronal progenitors may explain TF targeting by multiple miRNAs. These findings are consistent with a role for miRNAs helping to regulate the transition from the proliferative and migratory phenotype of neuronal progenitors into differentiating neurons. Confirmation of the role of miRNAs in the regulation of neurogenesis awaits functional studies using loss and gain of function experiments.

There were differentially regulated miRNAs identified in this study that did not have a negative correlation, and there were two miRNAs (miR-290, miR-218) that had a significant positive correlation with predicted target mRNAs. The lack of negative correlation could be caused by inaccuracies in miRNA target prediction or by a translational inhibition mechanism rather than RNA degradation, which would prevent detection with mRNA microarrays. The detection of miRNAs with a positive correlation between the miRNA and target mRNA (either both down-regulated or both being up-regulated) suggests some alternative indirect mechanisms or a positive regulatory role for miRNAs, as has been reported for some genes involved in cell cycle regulation [34].

## Conclusion

Understanding the molecular mechanisms that regulate cortical neurogenesis will require the identification of genes with the correct spatial and temporal expression patterns. In this study, we report the mRNA and miRNA expression profiles of the first neuronal progenitors in the developing telencephalon, and provide a catalog of TFs likely to play a role in telencephalon development. In addition, we show that the expression of many dynamically regulated miRNAs are negatively correlated with the expression of their predicted target mRNAs. The complexity of cortical development may require additional levels of gene regulation, and the miRNAs identified in this study are well positioned to regulate gene expression during the transition from neuronal progenitors into differentiated neurons.

## Methods

### Cell preparation and FACS

The animals used in this study were handled in accordance with an animal protocol approved by the National Institutes of Health IACUC. Telencephalic dissociates were prepared from time-pregnant Sprague-Dawley rats (Taconic, Hudson, NY), as previously described [35]. The age of embryos at each time point was confirmed using crown-rump length. Briefly, the whole telencephalon was microdissected and the tissue was dissociated using gentle trituration in papain. The dissociated cells were purified on a Percoll gradient. Telencephalon dissociates were labeled with tetanus-toxin fragment C (TnTx) (Sigma, St. Louis, MO) and biotinylated cholera-toxin B subunit (ChTx) (Roche, Indianapolis IN). TnTx was immunodetected with mouse IgG anti-TnTx and goat anti-mouse IgG conjugated with phycoerythrin (PE) and Cy5 tandem dye (Invitrogen, Carlsbad, CA). ChTx was detected with streptavidin conjugated with PE and Cy5 tandem dye (Invitrogen). The telencephalon dissociates were simultaneously labeled with a combination of mouse IgM anti-A2B5 (Chemicon, Temecula, CA) and anti-Jones (CDw60) (Sigma) and detected using goat anti-mouse IgM conjugated with PE (Jackson ImmunoResearch, West Grove, PA). Labeled cell suspensions positive for TnTx and ChTx, but negative for A2B5 and Jones were physically and rapidly sorted into collection tubes containing RNAlater (Ambion, Austin, TX) using a FACSVantageSE flow cytometer (Becton Dickinson, San Jose, CA).

### RNA extraction/labeling

Total RNA was extracted using the RNAeasy micro kit (Qiagen, Valencia, CA). The quality of total RNA was assessed using Agilent's Bioanalyzer microchip (Palo Alto, CA). Briefly, 100 nanograms of total RNA was amplified following Affymetrix's small sample labeling protocol. The protocol uses two rounds of reverse transcription and *in vitro *transcription with a biotin label being incorporated during the second round of *in vitro *transcription.

### mRNA microarrays

10 micrograms of biotin-labeled and fragmented cRNA was hybridized to Affymetrix Rat expression 230 2.0 microarrays (Affymetrix, Santa Clara, CA). Four independent biological replicates were included in each group. The microarrays were hybridized, washed and scanned according to Affymetrix standard protocols. The raw data was deposited in the Gene Expression Omnibus (GEO), [accession number GSE11334], at [36]. The quality of the microarray data was assessed by the consistency of the percent present calls, background signal, rawQ and noise. The data was imported into Genespring, (Agilent, Santa Clara, CA) for normalization and data analysis. Robust Multi-Array Analysis (RMA) was used for pre-processing of the raw CEL files, and the data were normalized using a global per chip normalization (normalized to the 50^th ^percentile) and per gene normalization (normalized to the median). A 2-fold difference in normalized expression in combination with a Welch *t-test *was used to identify differentially expressed transcripts. The *t-test *was performed without an assumption of equal variances, and Benjamini and Hochberg multiple testing correction was used with a false discovery rate of 0.05.

### miRNA microarrays

Total RNA from FACS-purified E11, E12 and E13 neuronal progenitors was purified using the mirVana miRNA Isolation Kit (Ambion) and used for hybridization with miRNA microarrays (LC Sciences, Houston, TX). Three independent biological replicates were included in each group. The raw data was deposited in the EMBL/EBI ArrayExpress database, [accession number E-MEXP-1596], at . Slides were scanned using the Axon GenePix 4000B microarray scanner (Axon Instruments, Union City, CA). The microarray images were background subtracted using a local regression method and normalized to the statistical mean of all detectable miRNAs.

### mRNA qRT-PCR

The LightCycler (Roche) was used to perform relative qRT-PCR. The cDNA was generated from total RNA isolated from neuronal progenitors purified as described in the cell preparation section. PCR primer pairs specific for seven TFs were designed to span one intron and to yield an approximately 200 bp product size. Melting curves were generated using the LightCycler analysis software to determine whether there were any spurious amplification products, and the PCR products were run on an agarose gel to confirm the presence of a single band. Beta-actin was chosen as a reference gene because it was expressed at equivalent levels in each group according to the microarray data and in preliminary qRT-PCR experiments. Relative qRT-PCR was used to determine the fold difference for the seven TFs [37]. In this method, the PCR efficiency is experimentally determined for each primer pair and calculated as efficiency = 10 ^(-1/slope) ^with the slope being determined using a dilution series and by the LightCycler analysis software. The difference in cycle threshold between E11 samples and E13 samples was normalized by dividing the difference in the cycle threshold for beta-actin in E11 and E13 cDNA samples.

### miRNA qRT-PCR

150 ng of total RNA from E13 neuronal progenitors was treated with Turbo DNase (Ambion) for 10 minutes at 37°C. Two independent biological replicates were used in the experiment. First strand synthesis was conducted using the Taqman Reverse Transcription Kit (Applied Biosystems, Foster City, CA). Real-time quantitative PCR was performed using a Taqman Array microRNA Panel v1.0 (Early Access) obtained from Applied Biosystems. PCR was performed on Applied Biosystems 7900HT Fast Real-Time PCR Systems using the Taqman 2X Universal PCR Master Mix. The primer sequences are available at .

### Immunohistochemistry

E13 sections prepared by Histoserve (Gaithersburg, MD) were permeabilized with 0.1% Triton X-100 for 10 minutes and blocked for 30 minutes with 10% normal donkey serum and 1% BSA. Anti-Cutl1 mouse IgG (Abcam, Cambridge, MA) was applied to sections for 2 hours at room temperature. The sections were washed three times for 5 minutes in PBS and detected with a donkey anti-mouse-IgG Alexa Fluor 594 for 1 hour. Vectashield with DAPI (Vector Lab, Burlingame, CA) was added prior to cover slipping. The sections were observed with an Olympus IX70 microscope and a 40× objective with a 0.6 numerical aperture. The images were captured with a Cool Snap digital camera (Photometrics, Tucson, AZ).

Neural cell phenotypes emerging at the onset of cortical neurogenesis were identified using a multi-color immunostaining protocol, as previously described [38]. Briefly, 8 μm thick sagittal sections of E12 rat telencephalon were immunolabeled for 1 hr at room temperature (RT) using the following mixture of primary antibodies: mouse IgG1 anti-nestin (Millipore, Billerica, MA), mouse IgG2a anti-proliferation cell nuclear antigen (PCNA, Millipore), mouse IgG2b anti-tubulin beta III (clone Tuj1, Sigma-Aldrich) and mouse IgM anti-CDw60 (clone Jones, Sigma-Aldrich). Primary immunoreactions were visualized after a 1 hr incubation at RT with the following mixture of Alexa Fluor (AF)-conjugated goat secondary antibodies: anti-mouse IgG1-Alexa Fluor-AF350, anti-mouse IgG2a-AF546, anti-mouse IgG2b-AF647 and anti-mouse IgM-AF488 (Invitrogen, Carlsbad, CA). The sections were imaged using the Axiovert 200 M fluorescence microscope (Carl Zeiss, Thornwood, NY) equipped with standard filter sets (Semrock, Rochester, NY) to detect the above AF fluorophores.

### Bioinformatics analysis

Correlation analysis of the miRNA and mRNA expression profiles was carried out using the lists of differentially expressed miRNAs and mRNAs. The predicted mRNA targets of each of the miRNAs that were differentially regulated were obtained using TargetScan version 4.0 [39]. The TargetScan identifier was converted to an Affymetrix probe set identifier using Affymetrix's Netaffx web tool [40]. A two-tailed Fisher's Exact Test was conducted for each differentially regulated miRNA to determine whether the number of predicted target mRNAs that were differentially regulated was higher than would be expected by chance (P < 0.05). The Fisher Exact test was conducted for each miRNA using both down-regulated and up-regulated mRNA gene lists. Gene ontology analysis was conducted using DAVID [41]. Network analysis was performed using Osprey [42].

## Abbreviations

CNS: central nervous system; ENP: early neuronal progenitor; LNP: late neuronal progenitor; NGP: neuroglial progenitor; GO: gene ontology; FACS: fluorescence-activated cell sorting; qRT-PCR: quantitative reverse transcription PCR; TF: transcription factor; miRNA: microRNA.

## Authors' contributions

JAN contributed to the study design, mRNA microarray experiments, validation experiments, data analysis and manuscript drafting. PL contributed to the study design, miRNA microarrays, validation experiments, data analysis, manuscript drafting. DM contributed to the study design, flow cytometry, immunohistochemistry, manuscript drafting. JLB contributed to study design and manuscript drafting. LDH contributed to study design and manuscript drafting. All authors read and approved the final manuscript.

## Supplementary Material

Additional file 1 **qRT-PCR validation of miRNA microarray expression data.** The expression of 12 miRNAs identified as up-regulated between E11 and E13 were confirmed in E13 neuronal progenitors. Click here for file

Additional file 2 **83 mRNAs encoding transcription factors are down-regulated in neuronal progenitors between embryonic day 11 and day 13.** Click here for file

Additional file 3 **Gene ontology analysis identifies 33 transcripts annotated with the GO term: regulation of progression through cell cycle.** Click here for file
