# Supplementary material for: Integrating microRNA and mRNA expression profiles of neuronal progenitors to identify regulatory networks underlying the onset of cortical neurogenesis
Source: BMC Neurosci. 2009 Aug 19;10:98. doi: 10.1186/1471-2202-10-98 (PMC2736963; doi:10.1186/1471-2202-10-98)
Supplement: Additional file 1 — qRT-PCR validation of miRNA microarray expression data. The expression of 12 miRNAs identified as up-regulated between E11 and E13 were confirmed in E13 neuronal progenitors. [file 1471-2202-10-98-S1.doc]

Additional file 1. qRT-PCR validation of miRNA microarray expression data. The expression of 12 miRNAs identified as up-regulated between E11 and E13 were confirmed in E13 neuronal progenitors.

| **microRNA** | **crossing point** |
| --- | --- |
| miR-100 | 28.5 |
| miR-124a | 30.5 |
| miR-125a | 27.0 |
| miR-125b | 26.5 |
| miR-181b | 29.0 |
| miR-181c | 33.0 |
| miR-218 | 28.4 |
| miR-376a | 33.0 |
| miR-7 | 32.5 |
| miR-9 | 23.5 |
| miR-99a | 28.5 |
| miR-99b | 27.0 |
