# Supplementary material for: Integrating microRNA and mRNA expression profiles of neuronal progenitors to identify regulatory networks underlying the onset of cortical neurogenesis
Source: BMC Neurosci. 2009 Aug 19;10:98. doi: 10.1186/1471-2202-10-98 (PMC2736963; doi:10.1186/1471-2202-10-98)
Supplement: Additional file 2 — 83 mRNAs encoding transcription factors are down-regulated in neuronal progenitors between embryonic day 11 and day 13. [file 1471-2202-10-98-S2.doc]

Additional file 2. 83 mRNAs encoding transcription factors are down-regulated in neuronal progenitors between embryonic day 11 and day 13.

| **Affymetrix ID** | **fold** | **Genebank** | **Gene** | **Gene Description** |
| --- | --- | --- | --- | --- |
| 1398503_at | 15.9 | AI575653 | Rax | retina and anterior neural fold homeobox |
| 1387306_a_at | 13.8 | NM_053633 | Egr2 | early growth response 2 |
| 1368308_at | 10.1 | NM_012603 | Myc | myelocytomatosis viral oncogene homolog (avian) |
| 1368711_at | 9.4 | NM_012743 | Foxa2 | forkhead box A2 |
| 1368998_at | 7.4 | NM_031737 | Nkx6-1 | NK6 transcription factor related, locus 1 (Drosophila) |
| 1382687_at | 7.2 | BE096569 | Six6 | sine oculis-related homeobox 6 hom. (Drosophila) (predicted) |
| 1393984_at | 7.1 | BF521686 |  | Forkhead box B1 (predicted) |
| 1387769_a_at | 5.5 | AF000942 | Id3 | Inhibitor of DNA binding 3 |
| 1368321_at | 4.9 | NM_012551 | Egr1 | early growth response 1 |
| 1376197_at | 4.6 | AW251860 | Tcf7 | transcription factor 7, T-cell specific (predicted) |
| 1386949_a_at | 4.3 | AJ132046 | Mta1 | metastasis associated 1 |
| 1389351_at | 3.7 | BM387864 |  | similar to FLI-LRR associated protein-1 |
| 1395557_at | 3.5 | BF565718 | Copeb | Core promoter element binding protein |
| 1388309_at | 3.3 | BG378885 | Hmga1 | high mobility group AT-hook 1 |
| 1367605_at | 3.3 | NM_022511 | Pfn1 | profilin 1 |
| 1374404_at | 3.3 | BI288619 | Jun | v-jun sarcoma virus 17 oncogene homolog (avian) |
| 1375043_at | 3.3 | BF415939 | Fos | FBJ murine osteosarcoma viral oncogene homolog |
| 1386823_at | 3.2 | BF545481 |  | iroquois homeobox protein 5 |
| 1392329_at | 3.2 | BI295531 |  | Homeodomain leucine zipper-encoding gene |
| 1384312_at | 3.2 | BF543574 | Irx1 | Iroquois related homeobox 1 (Drosophila) (predicted) |
| 1373530_at | 3.1 | AW913890 | Ccne1 | cyclin E |
| 1378692_at | 3.1 | BF564165 | Pax8 | Paired box gene 8 |
| 1380354_at | 3.1 | BF388953 | Znf206 | zinc finger protein 206 (predicted) |
| 1372564_at | 3.0 | AI411375 |  | Transcribed locus |
| 1378803_at | 3.0 | BF400968 | Nkx6-2 | NK6 transcription factor related, locus 2 (Drosophila) (predicted) |
| 1377702_at | 3.0 | BG380173 | Rb1 | Retinoblastoma 1 |
| 1398826_s_at | 3.0 | NM_139113 | Nr2f6 | nuclear receptor subfamily 2, group F, member 6 |
| 1391602_at | 3.0 | AW527113 | Drg1 | developmentally regulated GTP binding protein 1 (predicted) |
| 1369052_at | 2.9 | NM_133323 | Zfp111 | zinc finger protein 111 |
| 1388274_at | 2.9 | AA849035 | Bmyc | brain expressed myelocytomatosis oncogene |
| 1370376_a_at | 2.9 | AA866458 | Csda | cold shock domain protein A |
| 1387028_a_at | 2.8 | M86708 | Id1 | Inhibitor of DNA binding 1, helix-loop-helix protein (splice var) |
| 1368233_at | 2.8 | NM_031042 | Gtf2f2 | general transcription factor IIF, polypeptide 2 |
| 1368368_a_at | 2.8 | NM_032616 | Lisch7 | liver-specific bHLH-Zip transcription factor 7 |
| 1368175_at | 2.8 | NM_133620 | Zhx1 | zinc-fingers and homeoboxes 1 |
| 1387060_at | 2.7 | NM_031642 | Copeb | core promoter element binding protein |
| 1378032_at | 2.7 | AI176265 |  | Transcribed locus |
| 1390776_at | 2.7 | AI030203 | Irx3 | Iroquois related homeobox 3 (Drosophila) (predicted) |
| 1373421_at | 2.7 | BM392224 | Tgif | TG interacting factor (predicted) |
| 1398875_at | 2.7 | AI105044 |  | estrous-specific protein, 250 kDa |
| 1377827_at | 2.7 | AA851587 |  | SRF-dependent transcription regulation associated protein |
| 1389760_at | 2.6 | AA858786 | Rnf134 | ring finger protein 134 (predicted) |
| 1381404_at | 2.6 | AW524425 |  | Methyl-CpG binding domain protein 1 (predicted) |
| 1369733_at | 2.6 | NM_053357 | Ctnnb1 | catenin (cadherin associated protein), beta 1, 88kDa |
| 1374904_at | 2.6 | AI175048 | Six1 | sine oculis homeobox homolog 1 (Drosophila) |
| 1371188_a_at | 2.6 | AI407688 | Ubtf | upstream binding transcription factor, RNA polymerase I |
| 1389623_at | 2.5 | AA849092 | Atf1 | activating transcription factor 1 (predicted) |
| 1369067_at | 2.5 | NM_031628 | Nr4a3 | nuclear receptor subfamily 4, group A, member 3 |
| 1390390_at | 2.5 | BM386814 | Tcf20 | transcription factor 20 |
| 1368777_at | 2.5 | NM_022622 | Bard1 | BRCA1 associated RING domain 1 |
| 1371035_at | 2.5 | BE098763 | Gtf3a | general transcription factor III A |
| 1386041_a_at | 2.5 | BF288243 |  | Kruppel-like factor |
| 1367984_at | 2.4 | NM_019384 |  | CTD-binding SR-like rA1 |
| 1387442_at | 2.4 | NM_019137 | Egr4 | early growth response 4 |
| 1383108_at | 2.4 | BE120513 | Taf13 | TAF13 RNA polymerase II |
| 1385387_at | 2.3 | AA925143 | Nkx2-2 | NK2 transcription factor related, locus 2 (Drosophila) (predicted) |
| 1367926_at | 2.3 | NM_031851 | Phb | prohibitin |
| 1384783_at | 2.3 | BI289823 |  | zinc finger protein 161 |
| 1399048_at | 2.3 | BI274121 | Carm1 | coactivator-associated arginine methyltransferase 1 (predicted) |
| 1370300_at | 2.3 | AF061817 | Preb | prolactin regulatory element binding |
| 1386129_at | 2.3 | BF523624 | Ing1l | inhibitor of growth family, member 1-like (predicted) |
| 1376648_at | 2.3 | BI275570 | Mycn | v-myc myelocytomatosis viral related oncogene, (avian) |
| 1393998_at | 2.3 | BF408529 | Gabpb1 | GA repeat binding protein, beta 1 (predicted) |
| 1370917_at | 2.2 | AI172496 | Hsf1 | heat shock transcription factor 1 |
| 1380030_at | 2.2 | AW523520 | Znf593 | zinc finger protein 593 (predicted) |
| 1389537_at | 2.2 | AI502399 | Tcof1 | Treacher Collins Franceschetti syn. 1, homolog (predicted) |
| 1387624_at | 2.2 | NM_031777 | Usf1 | upstream transcription factor 1 |
| 1391311_at | 2.2 | AA965274 | Sox21 | SRY-box containing gene 21 (predicted) |
| 1372985_at | 2.2 | BG378167 | Znf444 | zinc finger protein 444 (predicted) |
| 1394039_at | 2.2 | BM382886 | Klf5 | Kruppel-like factor 5 |
| 1373772_at | 2.1 | AI179516 | Dnmt1 | DNA (cytosine-5-)-methyltransferase 1 |
| 1388089_a_at | 2.1 | AY050655 | Rnf4 | ring finger protein 4 |
| 1387098_at | 2.1 | NM_031772 | Rpo1-4 | RNA polymerase 1-4 |
| 1370421_a_at | 2.1 | AB029342 | Tip120B | TBP-interacting protein 120B |
| 1388912_at | 2.1 | AI237143 |  |  |
| 1370968_at | 2.1 | AA858801 | Nfkb1 | nuclear factor of kappa light chain gene enhancer in B-cells 1, |
| 1368877_at | 2.1 | NM_052798 | Znf354a | zinc finger protein 354A |
| 1373004_at | 2.1 | AA964764 | Dnajc2 | DnaJ (Hsp40) homolog, subfamily C, member 2 |
| 1374409_at | 2.1 | AW253760 | Taf6l | TAF6-like RNA polymerase II |
| 1370386_at | 2.0 | AB002406 | Ruvbl1 | RuvB-like protein 1 |
| 1368330_at | 2.0 | NM_053720 | Aatf | apoptosis antagonizing transcription factor |
| 1385803_at | 2.0 | BM388555 | Gtf2h2 | general transcription factor II H, polypeptide 2 (predicted) |
| 1373741_at | 2.0 | BM386451 | Pus1 | pseudouridine synthase 1 (predicted) |
