# Supplementary material for: Integrating microRNA and mRNA expression profiles of neuronal progenitors to identify regulatory networks underlying the onset of cortical neurogenesis
Source: BMC Neurosci. 2009 Aug 19;10:98. doi: 10.1186/1471-2202-10-98 (PMC2736963; doi:10.1186/1471-2202-10-98)
Supplement: Additional file 3 — Gene ontology analysis identifies 33 transcripts annotated with the GO term: regulation of progression through cell cycle. [file 1471-2202-10-98-S3.doc]

Additional file 3. Gene ontology analysis identifies 33 transcripts annotated with the GO term: regulation of progression through cell cycle.

| **Affymetrix ID** | **fold** | **Genebank** | **Gene** | **Gene Description** |
| --- | --- | --- | --- | --- |
| 1368308_at | 10.1 | NM_012603 | Myc | myelocytomatosis viral oncogene homolog (avian) |
| 1376425_at | 3.5 | BF420705 | Tgfb2 | Transforming growth factor, beta 2 |
| 1374404_at | 3.3 | BI288619 | Jun | v-jun sarcoma virus 17 oncogene homolog (avian) |
| 1373530_at | 3.1 | AW913890 | Ccne1 | cyclin E |
| 1369010_at | 3.1 | NM_053677 | Chek2 | protein kinase Chk2 |
| 1383519_at | 3.1 | BI294137 | Hk2 | Hexokinase 2 |
| 1377702_at | 3.0 | BG380173 | Rb1 | Retinoblastoma 1 |
| 1370427_at | 2.7 | L06238 | Pdgfa | platelet derived growth factor, alpha |
| 1390201_at | 2.7 | AA943817 |  | Ras-related protein RAP-1A |
| 1388953_at | 2.6 | AA892598 | Gnl3 | guanine nucleotide binding protein-like 3 (nucleolar) |
| 1372013_at | 2.6 | BG380285 | Ifitm1 | interferon induced transmembrane protein 1 (predicted) |
| 1368777_at | 2.5 | NM_022622 | Bard1 | BRCA1 associated RING domain 1 |
| 1382370_at | 2.5 | BE113938 | Ccnf | cyclin F |
| 1368947_at | 2.4 | NM_024127 | Gadd45a | growth arrest and DNA-damage-inducible 45 alpha |
| 1370297_at | 2.4 | U10188 | Plk1 | polo-like kinase 1 (Drosophila) |
| 1378028_at | 2.4 | BM384425 | Mad2l1 | MAD2 (mitotic arrest def., homolog)-like1 (predicted) |
| 1369935_at | 2.3 | NM_012766 | Ccnd3 | cyclin D3 |
| 1367926_at | 2.3 | NM_031851 | Phb | prohibitin |
| 1377064_at | 2.3 | AI602811 | Dusp6 | dual specificity phosphatase 6 |
| 1368125_at | 2.3 | NM_019229 | Slc12a4 | solute carrier family 12, member 4 |
| 1378282_at | 2.3 | BI290750 | Csnk2a2 | casein kinase II, alpha 2, polypeptide (predicted) |
| 1367697_at | 2.3 | NM_031020 | Mapk14 | mitogen activated protein kinase 14 |
| 1376084_a_at | 2.2 | AI385371 | Espl1 | extra spindle poles like 1 (S. cerevisiae) (predicted) |
| 1383752_at | 2.2 | BG375714 | Nol1 | nucleolar protein 1 (predicted) |
| 1398297_at | 2.2 | NM_021746 | Mapk12 | mitogen-activated protein kinase 12 |
| 1394077_at | 2.1 | BF548080 |  | ras homolog gene family, member E |
| 1397642_at | 2.1 | BM389108 | Rad50 | RAD50 homolog (S. cerevisiae) |
| 1388484_at | 2.1 | BI296084 | Ube2c | ubiquitin-conjugating enzyme E2C (predicted) |
| 1370345_at | 2.1 | L11995 | Ccnb1 | cyclin B1 |
| 1397409_s_at | 2.1 | BE113999 | Wee1 | wee 1 homolog (S. pombe) (predicted) |
| 1368330_at | 2.0 | NM_053720 | Aatf | apoptosis antagonizing transcription factor |
| 1370294_a_at | 2.0 | U05341 | Cdc20 | cell division cycle 20 homolog (S. cerevisiae) |
| 1368870_at | 2.0 | NM_013060 | Id2 | Inhibitor of DNA binding 2 |
